# S15 Promoting Physical Activity and Health in Sports Clubs: the what, the why and the how!

**DOI:** 10.1093/eurpub/ckae114.264

**Published:** 2024-09-26

**Authors:** Aurélie Van Hoye, Jan Seghers

**Affiliations:** Örebro University, Sweden; KU Leuven, Belgium

## Abstract

**Purpose:**

The symposium will discuss the relationship between health and sport, to enlighten current configurations, as well as on how to foster the relationships between the two concepts.

**Methods:**

Four presentations are foreseen in the symposium. First, Susanna Geidne (Örebro University, Sweden) will tackle the what by introducing four core concepts (health promotion as an outcome of sport, in sport, through sport and health promoting sport), their stakes and challenges in terms of application. Second, Jenifer Gothilander (Mälardalen University, Sweden) will explore the why by describing participations patterns in organised and unorganised sport among adolescents in Sweden. The two last presentation will highlight the how of physical activity and health promotion in organised sport. Third, Catherine O’Sullivan (World Athletics, Monaco) will showcase how physical activity is fostered among World Athletics activities. Fourth, Kevin Barros (Université de Lorraine, France) will present results of a literature review on coaches’ health promotion skills, strategies and training needs.

**Results:**

Participants will learn that health promotion in sport is not given and better understand the difference between fostering health through sport and being health promoting, how to adapt policies to tackle participants trends, how to best organise sport events and activities to foster active lifestyle and discover different strategies that coaches adopt to promote health based on their communication, motivation or management skills.

**Conclusion:**

Finally, Aurélie Van Hoye (University of Limerick, Ireland) and Jan Seghers (Katholieke Universiteit Leuven, Belgium) will engage the conversation with the audience on how to better root physical activity and health promotion by taking organised sport contexts into account.

